# The Ca^2+^ Channel Blocker Verapamil Inhibits the In Vitro Activation and Function of T Lymphocytes: A 2022 Reappraisal

**DOI:** 10.3390/pharmaceutics14071478

**Published:** 2022-07-15

**Authors:** José Ignacio Veytia-Bucheli, Den Alejandro Alvarado-Velázquez, Lourival Domingos Possani, Roberto González-Amaro, Yvonne Rosenstein

**Affiliations:** 1 Departamento de Medicina Molecular y Bioprocesos, Instituto de Biotecnología, Universidad Nacional Autónoma de México, Av. Universidad 2001, Cuernavaca 62210, Mexico; jose-ignacio.veytiabucheli@unamur.be (J.I.V.-B.); daav_1092@hotmail.com (D.A.A.-V.); lourival.possani@ibt.unam.mx (L.D.P.); 2 Laboratoire de Chimie Bio-Organique, Département de Chimie, Faculté des Sciences, Université de Namur, Rue de Bruxelles 615, 5000 Namur, Belgium; 3 Posgrado en Ciencias, Instituto de Investigación en Ciencias Básicas y Aplicadas, Universidad Autónoma del Estado de Morelos, Av. Universidad 1001, Cuernavaca 62209, Mexico; 4 Centro de Investigación en Ciencias de la Salud y Biomedicina, Universidad Autónoma de San Luis Potosí, Av. Sierra Leona 550, San Luis Potosí 78210, Mexico; rgonzale@uaslp.mx

**Keywords:** verapamil, Ca^2+^ channel blockers, immunosuppression, T cell activation, cytokine production

## Abstract

Ca^2+^ channel blockers (CCBs) are commonly used to treat different cardiovascular conditions. These drugs disrupt the intracellular Ca^2+^ signaling network, inhibiting numerous cellular functions in different cells, including T lymphocytes. We explored the effect of the CCB verapamil on normal human peripheral blood T cell activation, proliferation, and cytokine production. Cells were activated by ligating CD3 or CD3/CD28 in the presence or absence of verapamil, and the expression of activation-induced cell surface molecules (CD25, CD40L, CD69, PD-1, and OX40), cell proliferation, and cytokine release were assessed by flow cytometry. Verapamil exerted a dose-dependent inhibitory effect on the expression of all the activation-induced cell surface molecules tested. In addition, verapamil diminished T cell proliferation induced in response to CD3/CD28 stimulation. Likewise, the production of Th1/Th17 and Th2 cytokines was also reduced by verapamil. Our data substantiate a potent in vitro suppressive effect of verapamil on T lymphocytes, a fact that might be relevant in patients receiving CCBs.

## 1. Introduction

Ion channels allow the flux of ions across cell membranes and control critical physiological processes such as muscle contraction, nerve impulse, hormonal secretion, gene expression, proliferation, apoptosis, development, and cell migration [1]. Ca^2+^ ions are universal second messengers that drive these cellular responses by binding to numerous Ca^2+^-sensitive effector proteins that transduce the information encoded in the spatiotemporal dynamics of Ca^2+^ signaling.

Alterations in Ca^2+^ signaling are the cause of numerous diseases [2,3]. Ca^2+^ channel blockers (CCBs) such as amlodipine, diltiazem, nifedipine, and verapamil are widely used to treat hypertension, cardiac arrhythmias, angina pectoris, and the prophylaxis of migraine headaches. Blocking the voltage-gated L-type (slowly inactivating) Ca^2+^ channels in vascular smooth muscle results in vasodilation and decreased peripheral vascular resistance. In cardiac muscle cells, the inhibition of L-type Ca^2+^ channels produces negative chronotropic, dromotropic, and inotropic effects [4,5].

Although CCBs are considered safe and adverse reactions are uncommon, different conditions are associated with CCB therapy, including increased risk for viral infections, hyporeactivity in delayed hypersensitivity skin tests, low response to vaccination, and reduced in vitro lymphocyte proliferation [6,7,8]. Accordingly, CCB therapy has been associated with a diminished rate of graft rejection of solid organ transplants [9,10,11,12,13] as well as an increased risk of cancer in elderly patients [9,14].

The levels of intracellular Ca^2+^ have a central role in T cell function. The engagement of the T cell receptor (TCR) with its cognate antigen presented by antigen-presenting cells triggers the mobilization of Ca^2+^ from intracellular stores, followed by a sustained elevation dependent on extracellular Ca^2+^. Lasting Ca^2+^ fluxes are necessary to recruit to and retain in the nucleus isoforms of the nuclear factor of activated T cells (NFAT), and other transcription factors to control the clonal expansion, cell survival, differentiation, and production of effector molecules [2].

In humans, Ca^2+^ release-activated Ca^2+^ channel protein 1 (ORAI1) is the predominant contributor to the TCR-induced Ca^2+^ influx. However, additional Ca^2+^ permeable channels have been proposed to mediate Ca^2+^ influx in T cells, including certain voltage-gated Ca^2+^ (Ca_v_) channels, transient receptor potential (TRP) channels, and purinergic ionotropic (P2X) receptors (reviewed in [1,15,16]). Additionally, the K^+^ voltage-gated channel subfamily A member 3 (K_v_1.3) and the intermediate conductance Ca^2+^-activated K^+^ channel protein 4 (K_Ca_3.1) regulate the membrane potential through the efflux of K^+^ cations to the extracellular space and generate the electrochemical potential that drives the entry of Ca^2+^ through the plasma membrane during T cell activation [17,18]. The blockade of the ion channels involved in the Ca^2+^ signaling network results in impaired T cell activation and function [2].

As the specificity of CCBs for L-type Ca^2+^ channels is poor and unrelated channels are often affected by these drugs, this study aimed to further explore, in vitro, the putative immunosuppressive effect of verapamil, a widely used CCB, a phenomenon that might be relevant in vivo.

## 2. Materials and Methods

### 2.1. Blood Samples and T Cell Purification

This procedure was approved by the Bioethics Committee of the Instituto de Biotecnología. Buffy coats from anonymized healthy donors were obtained from the Centro Estatal de la Transfusión Sanguínea (Cuernavaca, Mexico). Peripheral blood mononuclear cells (PBMCs) were separated through Ficoll-Paque PLUS (GE Healthcare Bio-Sciences AB, Uppsala, Sweden) density gradient centrifugation. Cells were resuspended in RPMI-1640 medium (HyClone, GE Healthcare Life Sciences, Logan, UT, USA) supplemented with 2% heat-inactivated fetal calf serum (By Productos, Guadalajara, Mexico) and incubated in 100 mm tissue-culture treated polystyrene dishes (8 × 10^7^ cells/dish) at 37 °C in 5% CO_2_ overnight to promote adhesion of monocytes to the plastic surface and cell arrest. Non-adherent cells were recovered to purify T cells by magnetic cell sorting (negative selection) with the Pan T Cell Isolation Kit (Miltenyi Biotec GmbH, Bergisch Gladbach, Germany). Briefly, non-T cells were labeled with a monoclonal antibody cocktail (biotin-conjugated anti-CD14, -CD15, -CD16, -CD19, -CD34, -CD36, -CD56, -CD123, and -CD235a). Subsequently, the preparation was incubated with anti-biotin secondary antibodies conjugated with magnetic MicroBeads and transferred to a LD Column (Miltenyi Biotec GmbH) placed on a MidiMACS Separator (Miltenyi Biotec GmbH) permanent magnet. T cells were recovered by elution. Purity (CD3^+^ cells) was always > 95%, as determined by flow cytometry with PE anti-human CD3d antibody (1 μg/mL, clone 7D6, Caltag Laboratories, Burlingame, CA, USA).

### 2.2. T Cell Stimulation Assays

T cells (1 × 10^6^ cells/mL) in RPMI-1640 medium supplemented with 10% heat-inactivated fetal calf serum were stimulated in 48-well polystyrene cell culture plates (5 × 10^5^ cells/well). T cells were stimulated with anti-human CD3e mAb (clone OKT3, IgG2a, home purified) in the presence or absence of anti-human CD28 mAb (clone CD28.2, IgG1, BioLegend, San Diego, CA, USA). The anti-CD3e antibody was immobilized on the wells’ surface (100 μL/well of a 9.5 μg/mL antibody solution in phosphate-buffered saline (PBS)) by incubating the plates for 2 h at 37 °C. Wells were washed three times with PBS to remove unbound antibody, after which the cells were seeded, and soluble anti-CD28 antibody (2 μg/mL) was added to the cells and cross-linked with goat anti-mouse IgG1 antibodies (2 μg/mL) (SouthernBiotech, Birmingham, AL, USA). Where indicated, verapamil hydrochloride (Kener, Toluca, Mexico) was added at the indicated concentrations five minutes before the onset of stimulation. Plates were incubated at 37 °C in 5% CO_2_ for the indicated times.

### 2.3. Cytokine Production Profile

The supernatants from T cells activated as indicated above for 24 or 72 h were collected, and cytokines were measured using the LEGENDplex Human Th Cytokine Panel 13-plex (BioLegend) and flow cytometry. Samples were acquired in a BD FACSCanto II (BD Biosciences, San Jose, CA, USA) flow cytometer with the BD FACSDiva (version 6.1.3, BD Biosciences) software and analyzed with the LEGENDplex Data Analysis Software (BioLegend).

### 2.4. Flow Cytometry

For cell viability determination, cells were washed with PBS and incubated with 500 μL of a 1:100,000 dilution of the Fixable Viability Dye eFluor 780 (Life Technologies, Carlsbad, CA, USA) in PBS at 4 °C for 30 min in the dark. To evaluate cell proliferation, 2 × 10^7^ cells were labeled with 1 mL of 10 µM CFSE (Life Technologies, Eugene, OR, USA) in RMPI at 37 °C for 15 min in the dark and then washed with RPMI-1640 supplemented with 10% heat-inactivated fetal calf serum. Then, cell proliferation was assessed after 120 h of stimulation. For fluorescent antibody staining, cells were washed with PBS. Fc receptors were blocked with 100 μL of 10% human serum in FACS solution (PBS supplemented with 0.5% bovine serum albumin and 0.1% sodium azide) at 4 °C for 30 min, and cells were stained in a final volume of 150 μL with a master mix containing the following fluorescent antibodies: APC anti-human CD25 (1 μg/mL, clone BC96, BioLegend), PE anti-human CD69 (0.25 μg/mL, clone FN50, BioLegend), FITC anti-human CD40L/CD154 (1 μg/mL, clone 24–31, BioLegend), Pacific Blue anti-human PD-1/CD279 (1.25 μg/mL, clone EH12.2H7, BioLegend), and PerCP/Cy5.5 anti-human OX40/CD134 (2.5 μg/mL, clone Ber-ACT35, BioLegend). Cells were incubated for 30 min at 4 °C in the dark, washed with FACS solution to remove unbound antibodies, fixed with 2% paraformaldehyde in PBS, and stored at 4 °C until analysis. Samples were acquired on a BD FACSCanto II (BD Biosciences) flow cytometer with the BD FACSDiva (BD Biosciences) software and analyzed using the FlowJo (version 8.7, FlowJo, LLC, Ashland, OR, USA) software. The expression level of the different molecules was expressed as mean fluorescence intensity (MFI) or percentage of positive cells.

### 2.5. Statistical Analysis

GraphPad Prism 8.0.2 (GraphPad Software, San Diego, CA, USA) software was used for data analysis and graph creation. Data are shown as arithmetic mean + standard deviation of the mean (SD). The Shapiro–Wilk test was used to determine the normality of the data. For comparisons between two groups, paired Student’s *t*-test (parametric data) and Wilcoxon matched-pairs test (non-parametric data) were used. For comparisons among multiple groups, repeated-measures ANOVA followed by Tukey’s Multiple Comparison *post hoc* test (parametric data) and Friedman test followed by Dunn’s *post hoc* test (non-parametric data) were used. Statistical significance was set at *p* < 0.05.

## 3. Results

### 3.1. Verapamil Inhibits T Cell Activation and Proliferation

**Figure 1:**
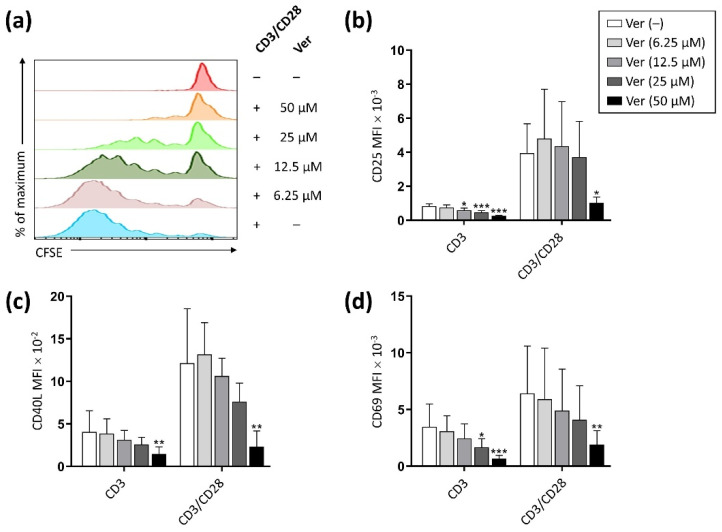
Verapamil inhibits T cell proliferation and activation in a dose-dependent manner. Purified T cells were stimulated (CD3 alone or CD3/CD28) in the absence or presence of different concentrations of verapamil (6.25–50 μM). (**a**) Cell proliferation was evaluated by CFSE dilution after 120 h of culture. Cells were stained after 24 h of culture for (**b**) CD25, (**c**) CD40L, and (**d**) CD69. Expression level of the activation-induced cell surface molecules is expressed as MFI. Data from 4 donors are shown as mean + SD. The significance of the pairwise comparisons between cells cultured without and with verapamil is indicated with asterisks (* *p* < 0.05, ** *p* < 0.01, *** *p* < 0.001).

We first evaluated the possible effect of verapamil on T cell proliferation. As shown in Figure 1a, verapamil exerted a dose-dependent inhibitory effect on the T lymphocyte proliferation in response to CD3 stimulation and CD28 costimulation, with almost complete inhibition at 50 μM. Furthermore, verapamil induced a dose-dependent reduction in the expression level of the activation-induced cell surface molecules CD25, CD40L, and CD69 (Figure 1b–d).

**Figure 2:**
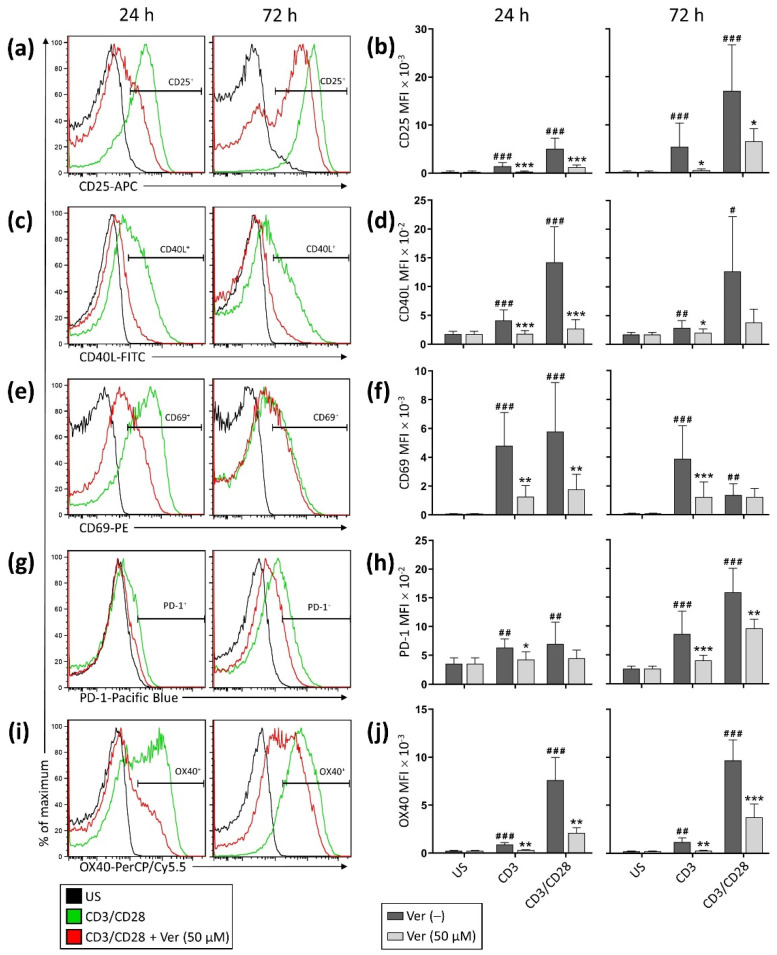
Verapamil decreases the expression of T cell activation-induced cell surface molecules. Purified T cells were stimulated (CD3 alone or CD3/CD28) in the absence or presence of verapamil (50 μM). Cells were stained for (**a**) CD25, (**c**) CD40L, (**e**) CD69, (**g**) PD-1, and (**i**) OX40 after 24 and 72 h of culture. A histogram of a representative donor for each activation-induced cell surface molecule is shown. (**b**,**d**,**f**,**h**,**j**) Expression level of the activation-induced cell surface molecules is expressed as MFI. Data from 4–12 donors are shown as mean + SD. The significance of the pairwise comparisons between unstimulated and stimulated (CD3 alone or CD3/CD28) cells is indicated with hash signs (^#^ *p* < 0.05, ^##^ *p* < 0.01, ^###^ *p* < 0.001). The significance of the pairwise comparisons between cells cultured without and with verapamil is indicated with asterisks (* *p* < 0.05, ** *p* < 0.01, *** *p* < 0.001).

Additional experiments showed that along with inhibiting the expression of CD25, CD40L, and CD69, verapamil inhibited the expression of other activation-induced cell surface molecules, namely PD-1 and OX40 (Figure 2). These results were observed following an incubation period of 24 or 72 h (data shown consider the MFI). Comparable results were observed when the data were expressed as the percentage of lymphocytes expressing the activation-induced cell surface molecules (Figure S1).

Interestingly, when cells were stimulated through CD3/CD28 as compared to only CD3, the inhibitory effect of verapamil was less profound, consistent with CD28 recruiting additional signaling pathways that bypass the TCR-activated Ca^2+^-dependent events inhibited by verapamil, partially rescuing T cell function [19].

Cell viability was not affected in the presence of verapamil at the end of the 24 h activation period (Figure S2). However, after 72 h verapamil significantly decreased cell viability, particularly in unstimulated cells, reflecting the importance of the Ca^2+^ signaling network in the survival of quiescent and activated T cells.

### 3.2. Verapamil Impairs the Production of Cytokines

**Figure 3:**
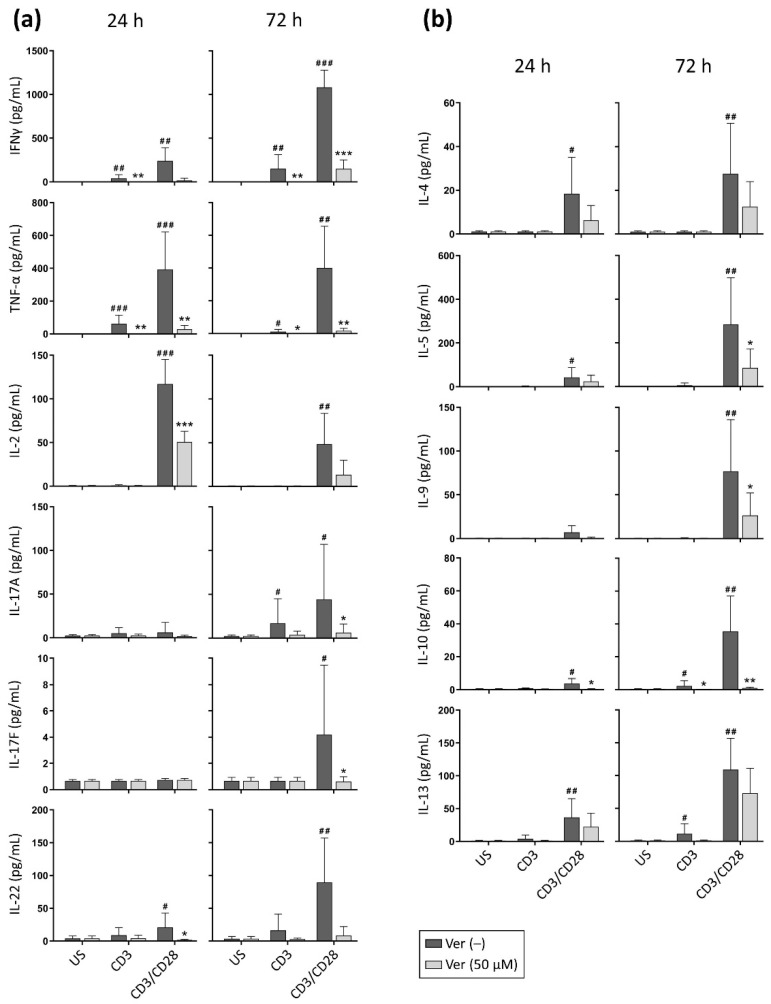
Verapamil decreases the production of Th1/Th17 and Th2 cytokines by T cells. Purified T cells were stimulated (CD3 alone or CD3/CD28) in the absence or presence of verapamil (50 μM). After 24 and 72 h of culture, cell supernatants were collected, and (**a**) Th1/Th17 (IFNγ, TNF-α, IL-2, -17A, -17F, and -22) and (**b**) Th2 (IL-4, -5, -9, -10, and -13) cytokines were quantified with a multiplex assay. Data from 6–12 donors are shown as mean + SD. The significance of the pairwise comparisons between unstimulated and stimulated (CD3 alone or CD3/CD28) cells is indicated with hash signs (^#^ *p* < 0.05, ^##^ *p* < 0.01, ^###^ *p* < 0.001). The significance of the pairwise comparisons between cells cultured without and with verapamil is indicated with asterisks (* *p* < 0.05, ** *p* < 0.01, *** *p* < 0.001).

T lymphocytes produce a variety of cytokines that play a central role in the final configuration of an immune response [20]. Since the intracellular Ca^2+^ concentration has a central role in the recruitment of several transcription factors (e.g., NFAT isoforms), promoting the expression of different cytokine genes, we evaluated whether verapamil altered cytokine production following T cell stimulation. As expected, T lymphocytes stimulated through CD3 produced small amounts of the cytokines tested (Figure 3); however, a significant decrease in IFNγ, TNF-α, and IL-10 in the media could be detected when verapamil was present.

When engaging CD3 and CD28, the cells released considerable quantities of the cytokines of the multiplex detection kit, except IL-6 or IL-21, which were not detected under our experimental conditions. In the presence of verapamil, a noticeable and significant decrease in IFNγ, TNF-α, IL-2, -5, -9, -10, -17A, -17F, and -22 concentration was detected in the media (Figure 3). IL-4 and -13 were also reduced in the presence of verapamil, although this was not statistically significant due to interindividual variation.

As T cell viability is not compromised after 24 h of culture in the presence of verapamil (Figure S2), and T cell division begins 40–50 h after stimulation [21], the mere inhibitory effect of verapamil on T cell activation could explain the decrease in cytokines in the media at the 24 h time-point. However, the reduction detected at the 72 h time-point reflects the combination of the inhibitory effect of verapamil on T cell activation, the reduced proliferation (Figure 1), and reduced cell viability (Figure S2). Our experiments do not allow us to elucidate whether the inhibitory effect of verapamil on cytokine production could be due, at least in part, to the blockade of lymphocyte proliferation. However, consistent with our results, a study evaluating the effects of verapamil analogues on the growth of *Mycobacterium tuberculosis* [22] reports that concentrations of verapamil as low as 6 μM inhibited the expansion of *M. tuberculosis*-specific T cells as well as IFNγ production by proliferating cells.

Overall, in vitro data (Figure 3) indicate that verapamil impairs the production of Th1/Th17 (IFNγ, TNF-α, IL-2, -17A, -17F, and -22) and Th2 (IL-4, -5, -9, -10, and -13) cytokines by stimulated T lymphocytes. Similar to the effect on the expression of activation-induced cell surface molecules, verapamil impaired cytokine production to a lesser extent when cells were costimulated, as the CD3/CD28 stimulus was more refractory to the inhibitory effects of verapamil, partially rescuing T cell function.

## 4. Discussion

Ca^2+^ signaling is tightly controlled during human T cell activation. While ORAI1 channels are the main Ca^2+^ entry pathway for a sustained increase in cytoplasmic Ca^2+^ concentrations, other ion channels such as K_v_1.3, K_Ca_3.1, Ca_v_, TRP channels, and P2X receptors have been proposed to modulate Ca^2+^ influx in T cells as well [1,15,16]. Ca^2+^ binds to ubiquitous Ca^2+^ sensors, including calmodulin (CaM), regulating protein kinases/phosphatases that activate transcription factors such as NFAT, cAMP-responsive element-binding protein (CREB), myocyte-specific enhancer factor 2 (MEF2), and nuclear factor-κB (NF-κB) isoforms. These transcription factors modulate the transcription of genes involved in cell proliferation, differentiation, cytokine production, survival, and cell death, thus placing the control of Ca^2+^ fluxes at the center of the T cell decision-making process [16,23].

Verapamil is a CCB commonly used to treat cardiovascular disorders. By inhibiting Ca^2+^ influx through L-type Ca^2+^ channels, CCBs promote cardiac and vascular smooth muscle cell relaxation [4,5]. However, these drugs are promiscuous ion channel blockers and have pleiotropic effects, as their targets are widely distributed [24]. As T cells are increasingly used for immunotherapy, one of the current challenges is understanding the role of the different molecules that participate in T cell activation and differentiation. The main goal of this investigation was to expand the available information regarding the immunosuppressive effect of verapamil on human T lymphocytes.

T cell activation requires the coordinated signals from the TCR and co-receptor molecules such as CD28 to transit to an effector cell phenotype. As the expression of CD25, CD40L, CD69, PD-1, and OX40 is Ca^2+^-dependent [25,26], we evaluated the effect of verapamil on the expression of these activation-induced cell surface molecules in CD3/CD28-stimulated T cells. These molecules regulate essential processes during the T cell response, such as the expression of co-stimulatory molecules, cytokine secretion, antibody isotype switching, the acquisition of an effector/memory phenotype, the regulation of the T cell migration pattern, cell survival, proliferation, promotion of tolerance, and return to immune homeostasis [27,28,29,30,31].

Under our experimental conditions, verapamil profoundly affected T cell activation. We showed that the addition of verapamil to purified human peripheral blood T lymphocytes at the onset of T cell activation resulted in a pronounced inhibition of CD25, CD40L, CD69, PD-1, and OX40 expression, likely resulting from the lack of activation of Ca^2+^-dependent transcription factors to stimulate gene transcription [25,32]. The recruitment and prolonged presence of Ca^2+^-dependent transcription factors in the nucleus is also indispensable for the transcription of cytokine genes [25,26], prompting us to evaluate the effect of verapamil on cytokine production. Accordingly, and consistent with previous reports [33,34,35,36,37,38,39,40,41,42,43], T cells also produced fewer Th1/Th17 and Th2 cytokines when in the presence of verapamil.

Interestingly, the verapamil-induced inhibition was more pronounced when only the TCR was engaged, as the simultaneous ligation of CD28 and the TCR partially bypassed this inhibition, likely due to the activation of alternative signaling pathways that do not entirely rely on sustained Ca^2+^ fluxes.

These results are consistent with previous descriptions of the immunomodulatory effect of verapamil on human T cells, whereby verapamil was reported to inhibit Ca^2+^ entry, ATP production, CD25 expression, the accumulation of inositol phosphates, the cell cycle progression, and the uptake of precursor molecules for the synthesis of proteins, RNA and DNA. Verapamil was also shown to impair the generation and function of cytotoxic T cells and the remodeling of T cell cytoskeleton, chemotaxis, motility, and transmigration [33,34,36,37,38,39,40,41,44,45,46,47,48,49,50,51,52,53,54,55,56]. Together with these reports, our data suggest that verapamil likely exerts a downregulatory effect on the different T cell subsets and could act as a broad-spectrum immunosuppressive molecule.

It is worth mentioning that the data we report herein were obtained within verapamil concentrations of ~10–50 µM. While the ORAI1 channels are resistant to verapamil [24], this range tallies with the concentrations required to inhibit other ion channels that modulate Ca^2+^ influx in human T cells such as K_Ca_3.1 (IC_50_ = 28 μM [17]), K_v_1.3 (IC_50_ = 8 μM [57]), Ca_v_ (IC_50_ ~10 μM for L-type [58], and ~20 μM for T-type channels [59,60]), as well as the P-glycoprotein (≥2 μM [42]). Human T cells also express significant levels of P-glycoprotein, an ATP-dependent transporter that participates in the non-specific transmembrane transport of metabolites, xenobiotics, and endogenous peptides/proteins. Inhibiting P-glycoprotein activity by monoclonal antibodies or small molecules such as verapamil results in a profound suppression of cytokine release from activated PBMCs [42], adding a deficient transmembrane cytokine transport to the verapamil’s overall T cell immunomodulatory effect. The possibility that these concentrations of verapamil affect additional molecular targets should not be excluded.

As a lipophilic molecule, verapamil is widely distributed from plasma to body tissues. In a rat animal model, following a single intraperitoneal injection of verapamil (30 mg/kg), peak plasma concentrations were around 1.5 μM, while 15 to 85 times higher amounts were found in organs such as lungs, liver, kidneys, and heart [61]. In humans, wide variations in the disposition of verapamil and drug accumulation can occur between individuals during a multiple-dose oral regime. Considering that with standard doses, peak plasma concentrations of 0.1–2.0 μM are reached [62], concentrations within the immunosuppressive range could be expected in tissues undergoing an immune response.

Verapamil is marketed as a racemic mixture of equal proportions of the S(–)- and R(+)-verapamil enantiomers. Although the S(–) enantiomer is more potent at inhibiting Ca^2+^ uptake in human mitogen-stimulated lymphocytes, both enantiomers have been reported to be equally potent suppressors of T cell function [39], suggesting that verapamil’s immunosuppressive effects may be, at least partially, independent of the inhibition of transmembrane Ca^2+^ fluxes.

Although obtained in vitro, with total T cells, our results reinforce previous data suggesting that verapamil would be expected to act on different T cell subsets, disabling T cell responses. Furthermore, verapamil and other CCBs are known to compromise other cells important for the generation of an immune response such as monocytes/macrophages [63], neutrophils [63,64,65,66], NK cells [33,39,46,67,68], B cells [69,70,71], and endothelial cells [53,54,72]. Overall, our data underpin a potent suppressive effect of verapamil on T lymphocytes, a phenomenon that might be relevant for patients receiving CCBs. In some cases (e.g., patients requiring immunosuppressive therapy), this phenomenon might be considered a desirable effect. In contrast, in others (e.g., patients with arterial hypertension without other diseases), this effect should be avoided or, at least, taken into account.

## Data Availability

The data presented in this study are available within this article.

## Supplementary Materials

The following supporting information can be downloaded at: https://www.mdpi.com/article/10.3390/pharmaceutics14071478/s1. Figure S1: Verapamil decreases the percentage of cells expressing the activation-induced cell surface molecules; Figure S2: Verapamil compromises T cell viability during long culture periods.
