# Supplementary material for: The Ca2+ Channel Blocker Verapamil Inhibits the In Vitro Activation and Function of T Lymphocytes: A 2022 Reappraisal
Source: Pharmaceutics. 2022 Jul 15;14(7):1478. doi: 10.3390/pharmaceutics14071478 (PMC9324055; doi:10.3390/pharmaceutics14071478)
Supplement: Supplementary file 1 [file pharmaceutics-14-01478-s001.zip › pharmaceutics-1749857-supplementary.pdf]

Supplementary Materials

The Ca^{2+} Channel Blocker Verapamil Inhibits the In Vitro Activation and Function of T Lymphocytes: A 2022 Reappraisal

José Ignacio Veytia-Bucheli [1,2], Den Alejandro Alvarado-Velázquez [1,3], Lourival Domingos Possani [1], Roberto González-Amaro [4] and Yvonne Rosenstein [1,*]

[1] Departamento de Medicina Molecular y Bioprocesos, Instituto de Biotecnología, Universidad Nacional Autónoma de México, Av. Universidad 2001, Cuernavaca 62210, Mexico; jose-ignacio.veytiabucheli@unamur.be (J.I.V.-B.); daav_1092@hotmail.com (D.A.A.-V.); lourival.possani@ibt.unam.mx (L.D.P.)

[2] Laboratoire de Chimie Bio-Organique, Département de Chimie, Faculté des Sciences, Université de Namur, Rue de Bruxelles 615, 5000 Namur, Belgium

[3] Posgrado en Ciencias, Instituto de Investigación en Ciencias Básicas y Aplicadas, Universidad Autónoma del Estado de Morelos, Av. Universidad 1001, Cuernavaca 62209, Mexico

[4] Centro de Investigación en Ciencias de la Salud y Biomedicina, Universidad Autónoma de San Luis Potosí, Av. Sierra Leona 550, San Luis Potosí 78210, Mexico; rgonzale@uaslp.mx

* Correspondence: yvonne.rosenstein@ibt.unam.mx; Tel.: +52-(777)-329-1606

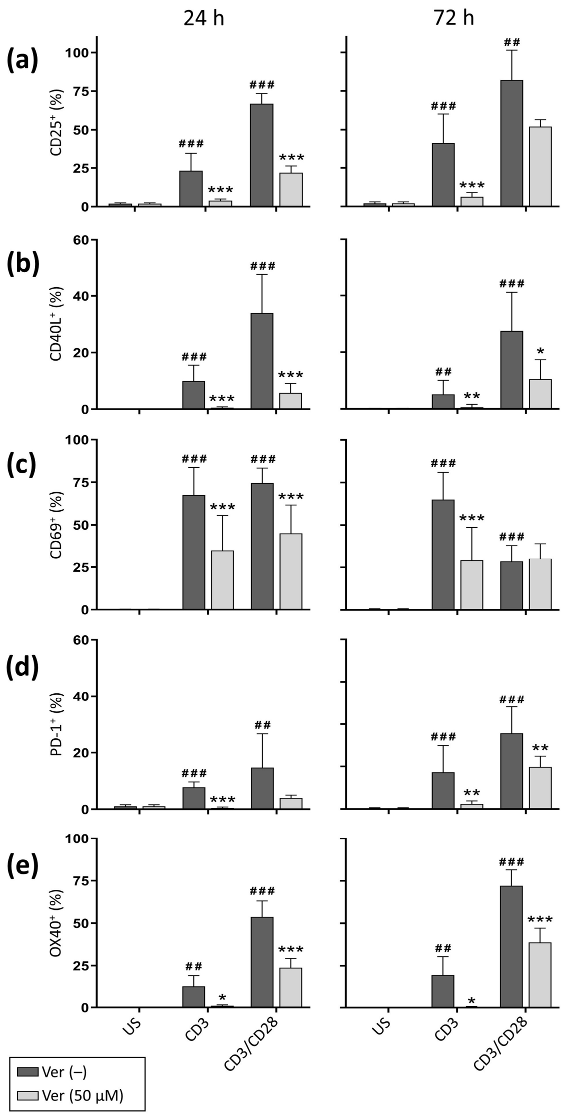

Figure S1. Verapamil decreases the percentage of cells expressing the activation-induced cell surface molecules. Purified T cells were stimulated (CD3 alone or CD3/CD28) in the absence or presence of verapamil (50 μM), and stained for CD25, CD40L, CD69, PD-1, and OX40 after 24 and 72 h of culture. (**a**–**e**) Proportion of cells expressing a particular activation-induced cell surface molecule (percentage of positive cells, P). Data from 4–12 donors are shown as mean + SD. The significance of the pairwise comparisons between unstimulated and stimulated (CD3 alone or CD3/CD28) cells is indicated with hash signs (## $p < 0.01$, ### $p < 0.001$). The significance of the pairwise comparisons between cells cultured without and with verapamil is indicated with asterisks (* $p < 0.05$, ** $p < 0.01$, *** $p < 0.001$).

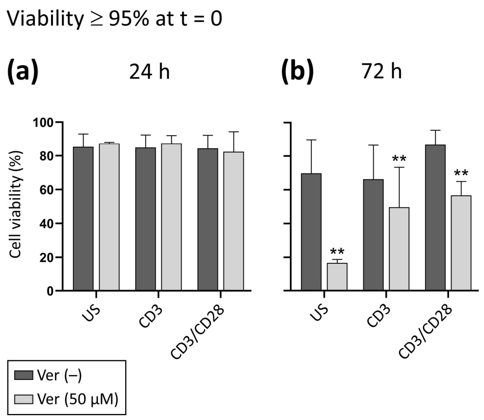

Figure S2. Verapamil compromises T cell viability during long culture periods. Cell viability of purified T cells was assessed after (**a**) 24 h and (**b**) 72 h of culture. T cells were stimulated (CD3 alone or CD3/CD28) in the absence or presence of verapamil (50 μM). Positive staining with the Fixable Viability Dye eFluor 780 was considered as a cell death marker. Data from 6 donors are shown as mean + SD. The significance of the pairwise comparisons between cells cultured without and with verapamil is indicated with asterisks (** $p < 0.01$).
